# Supplementary material for: Recruitment of Mre11 to recombination sites during meiosis
Source: bioRxiv. 2025 Jul 8:2025.07.08.663703. Preprint. [Version 1] doi: 10.1101/2025.07.08.663703 (PMC12265730; doi:10.1101/2025.07.08.663703)
Supplement: Supplement 1 [file NIHPP2025.07.08.663703v1-supplement-1.pdf]

bioRxiv preprint doi: https://doi.org/10.1101/2025.07.08.663703; this version posted July 8, 2025. The copyright holder for this preprint (which was not certified by peer review) is the author/funder, who has granted bioRxiv a license to display the preprint in perpetuity. It is made available under aCC-BY 4.0 International license.

Supplementary Information:

Recruitment of Mre11 to recombination sites during meiosis.

Priyanka Priyadarshini[1], Mahesh Survi[1], Wael El Yazidi Mouloud[2,3], Regina Bohn[4], Steven Ballet[2], Neil Hunter[4,5], Alexander N. Volkov[3,6] and Corentin Claeys Bouuaert[1,*]

[1] Louvain Institute of Biomolecular Science and Technology, Université catholique de Louvain, 1348 Louvain-La-Neuve, Belgium.
[2] Research Group of Organic Chemistry, Vrije Universiteit Brussel (VUB), Pleinlaan 2, 1050 Brussels, Belgium.
[3] Jean Jeener NMR Centre, Vrije Universiteit Brussel (VUB), Pleinlaan 2, 1050 Brussels, Belgium.
[4] Howard Hughes Medical Institute, University of California Davis, Davis, CA 95616, USA.
[5] Department of Microbiology & Molecular Genetics, University of California Davis, Davis, CA 95616, USA.
[6] VIB-VUB Center for Structural Biology, VIB, Pleinlaan 2, 1050 Brussels, Belgium.

* Correspondence to corentin.claeys@uclouvain.be

Supplementary Figures:
Figure S1: Properties of Mre11 nucleoprotein condensates.
Figure S2: Properties of MRX nucleoprotein condensates.
Figure S3: The C-terminal IDR of Mre11 is required for condensation.
Figure S4: Mre11 foci formation and expression during meiosis.
Figure S5: AlphaFold2 models of Mre11-Mer2 and conservation of Mre11-LLK and Mer2-EQEK residues.
Figure S6: Analysis of Mer2 – Mre11 interaction mutants.
Figure S7: The Mre11 C-terminus contains a novel SUMO-interaction motif.
Figure S8: NMR analysis of Smt3 bound to wild type and mutant SIM3 peptides.
Figure S9: ITC analysis of Smt3 binding to wild-type or mutant SIM3 peptides.
Figure S10: Protein expression and meiotic progression of Mre11 SIM3 mutants.

Supplementary Tables:
Table S1. Oligonucleotides used in this study.
Table S2. Plasmids used in this study.
Table S3. Synthetic peptides used in this study.
Table S4. Yeast strains used in this study.
Table S5. Protein sequences used for AlphaFold modeling.

bioRxiv preprint doi: https://doi.org/10.1101/2025.07.08.663703; this version posted July 8, 2025. The copyright holder for this preprint (which was not certified by peer review) is the author/funder, who has granted bioRxiv a license to display the preprint in perpetuity. It is made available under aCC-BY 4.0 International license.

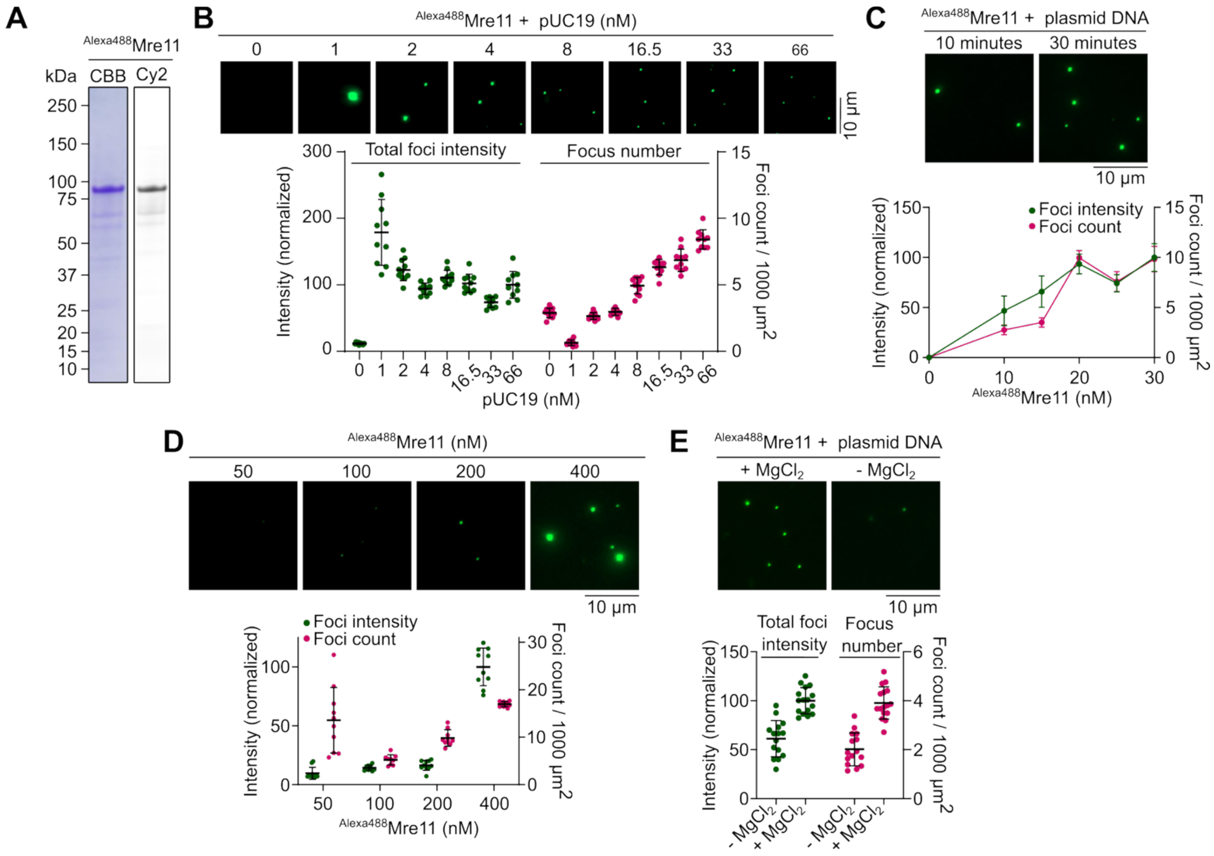

Figure S1: Properties of Mre11 nucleoprotein condensates.

(A) SDS-PAGE of Alexa488-labelled Mre11 stained with Coomassie Brilliant Blue (CBB) (left) and visualized with a Cy2 filter (right). **(B)** Effect of plasmid DNA (pUC19) concentration on ^{Alexa488}Mre11 condensate, visualized by epifluorescence microscopy. Quantification shows total fluorescence intensity (green) in a field of view normalized to the highest DNA concentration, and total number of foci per 1000 μm^2 (magenta). Error bars represent mean ± SD from 10-11 fields of view. **(C)** Time-dependent change in Mre11 condensate assembly. Reactions contained 400 nM ^{Alexa488}Mre11, 5.7 nM plasmid DNA and 5% PEG. Samples were collected at the indicated time points, immediately placed on a glass slide, covered with coverslip, and imaged. Foci intensities are normalized to the mean of the sample drawn at 30 minutes post-incubation. Error bars represent mean ± SD from 10-15 fields of view. **(D)** Effect of Mre11 concentration on condensation in presence of plasmid substrate and 5% PEG. Foci intensities are normalized to the mean of the sample with 400 nM Mre11. Error bars represent mean ± SD from 10 fields of view. **(E)** Effect of presence of divalent cation (5 mM $MgCl_2$) on Mre11 condensates assembled in presence of 5.7 nM pUC19. Foci intensities are normalized to reaction in the presence of magnesium. For experiment performed without magnesium (E), 5 mM EDTA was included in the reaction.

bioRxiv preprint doi: https://doi.org/10.1101/2025.07.08.663703; this version posted July 8, 2025. The copyright holder for this preprint (which was not certified by peer review) is the author/funder, who has granted bioRxiv a license to display the preprint in perpetuity. It is made available under aCC-BY 4.0 International license.

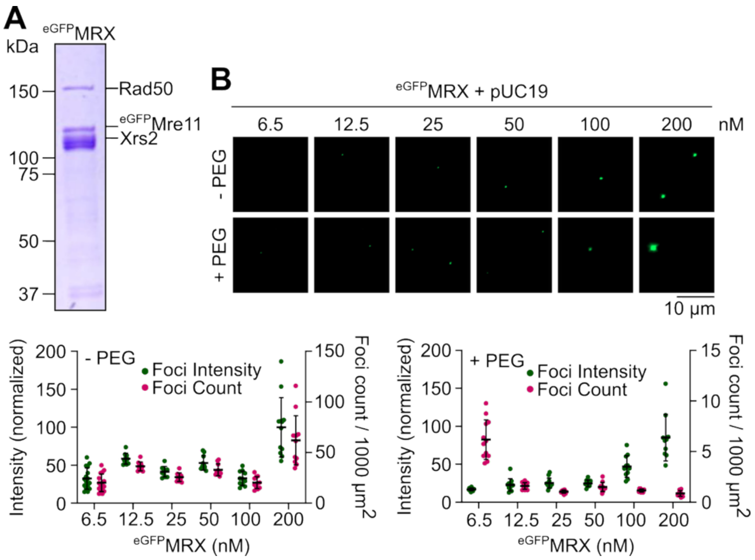

Figure S2: Properties of MRX nucleoprotein condensates.
(A) SDS-PAGE of eGFP-tagged Mre11-Rad50-Xrs2 (MRX) complex stained with Coomassie Brilliant Blue. **(B)** Effect of MRX concentration on nucleoprotein condensation in the presence or absence of 5% PEG. Foci intensities are normalized to the mean of the sample with 400 nM Mre11.

bioRxiv preprint doi: https://doi.org/10.1101/2025.07.08.663703; this version posted July 8, 2025. The copyright holder for this preprint (which was not certified by peer review) is the author/funder, who has granted bioRxiv a license to display the preprint in perpetuity. It is made available under aCC-BY 4.0 International license.

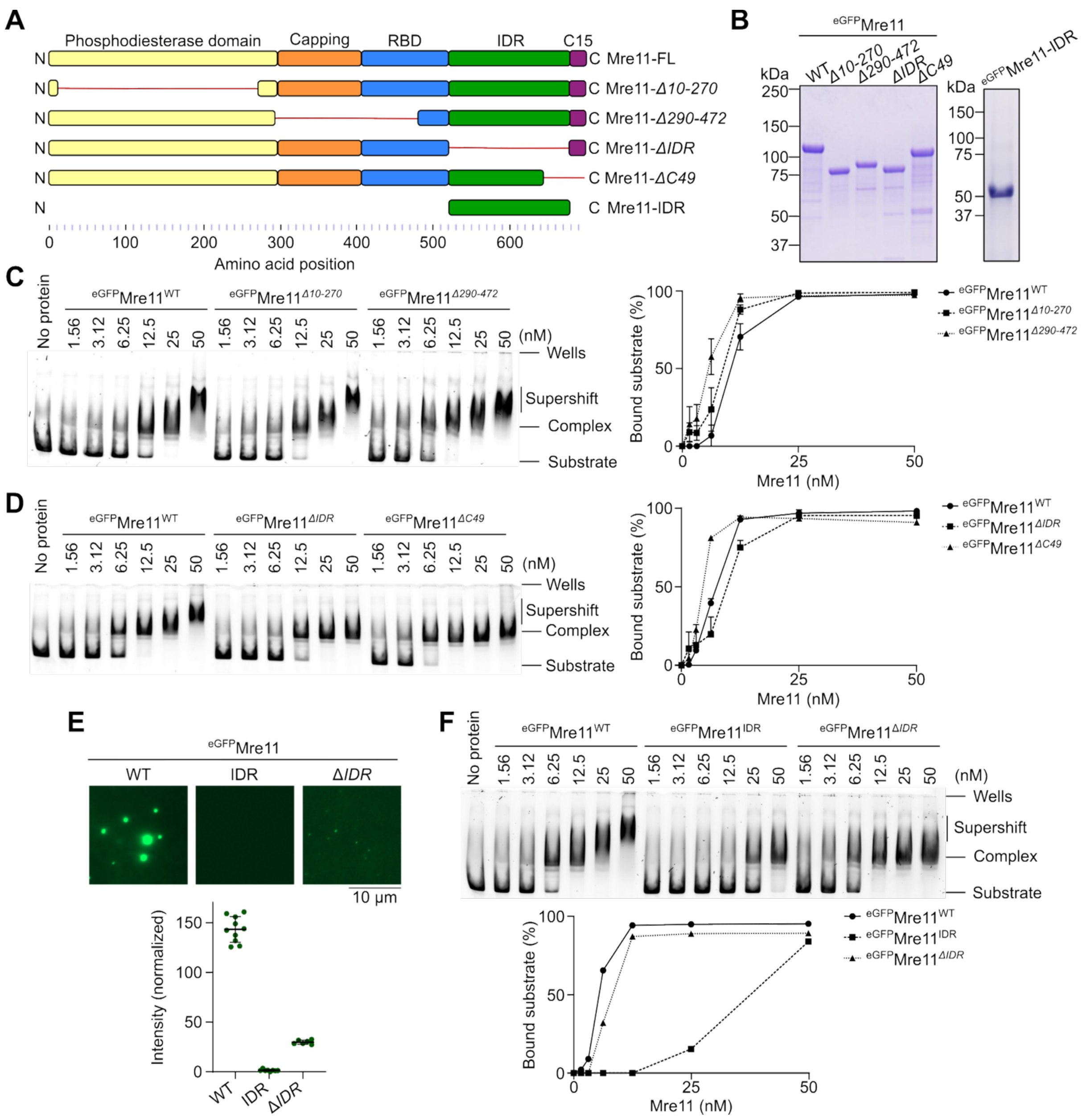

Figure S3: The C-terminal IDR of Mre11 is required for condensation.
(A) Cartoon diagram of full-length and truncated Mre11. IDR ranges from residues 524-677. Red line indicates truncated regions. **(B)** SDS-PAGE of purified WT and truncated ^{eGFP}Mre11. **(C, D)** Effect of Mre11 truncations on plasmid DNA binding analyzed by gel shift assay. Error bars in C and D show ranges from two independent experiments. **(E)** *In vitro* condensation analysis of eGFP-tagged Mre11, Mre11-IDR (524-677), and Mre11-*ΔIDR* (*Δ524-677*). Foci intensities are normalized to the mean of wild-type ^{eGFP}Mre11. Error bars represent mean ± SD from 6-10 fields of view. **(F)** Plasmid DNA binding of eGFP-tagged Mre11-IDR in comparison with Mre11 and Mre11-*ΔIDR* analyzed by gel shift assay.

bioRxiv preprint doi: https://doi.org/10.1101/2025.07.08.663703; this version posted July 8, 2025. The copyright holder for this preprint (which was not certified by peer review) is the author/funder, who has granted bioRxiv a license to display the preprint in perpetuity. It is made available under aCC-BY 4.0 International license.

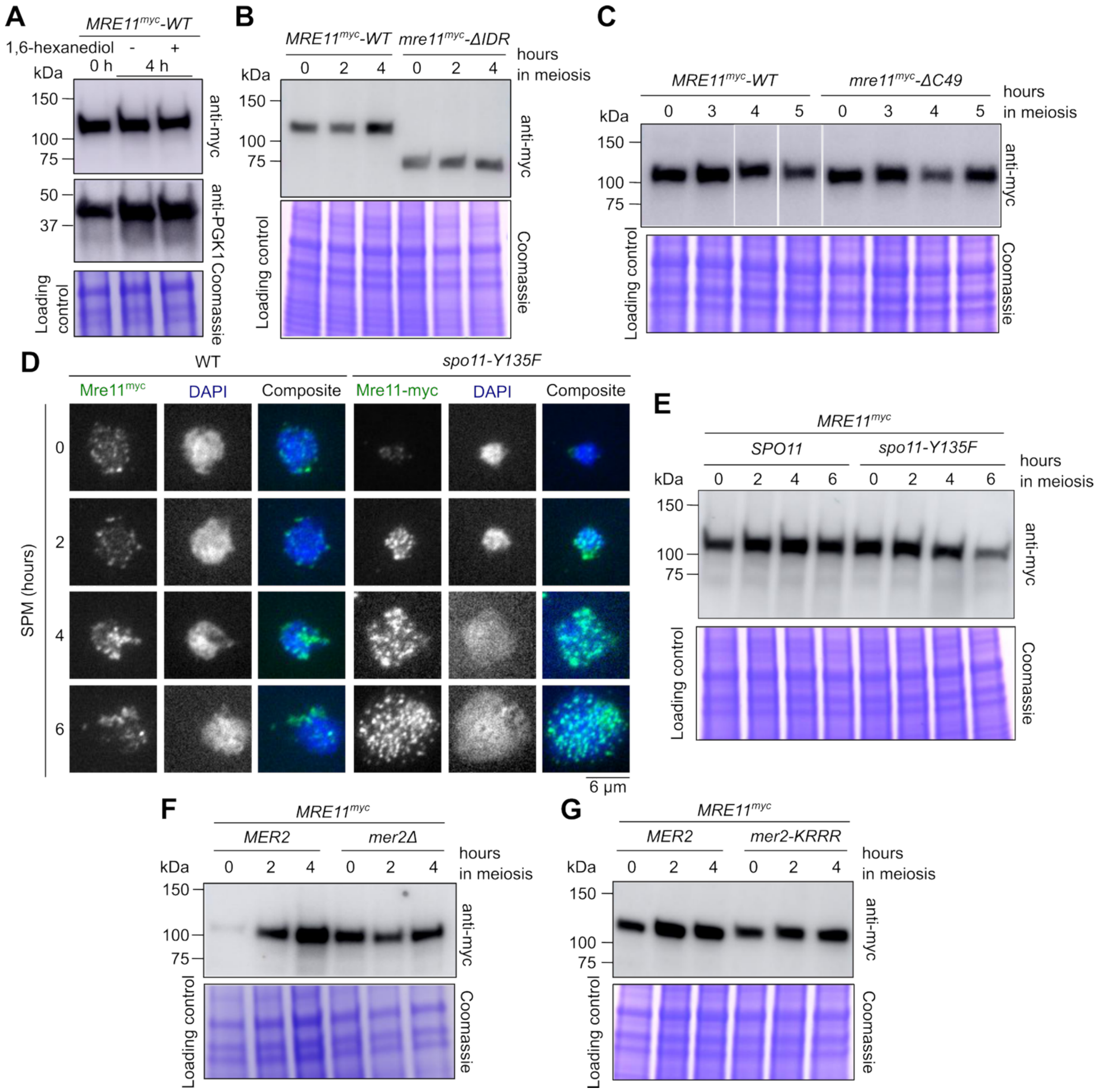

Figure S4: Mre11 foci formation and expression during meiosis.
(A, B, C, E, F, G) Western blot analysis of meiotic extracts of Mre11^{myc} in (A) 1,6-hexanediol-treated Mre11^{myc} strains harvested 4 hours after transferring to SPM, (B) wild type and Mre11-*ΔIDR* strains, (C) wild type and Mre11-*ΔC49* strains, (E) wild type and *spo11-Y135F* strains, (F) wild-type and *mer2Δ* strains, and (G) wild-type and *mer2-KRRR* strains. Coomassie-stained SDS-PAGE gels and anti-PGK1 Wester blots (panel 1) serve as loading controls. In panel C, all samples were loaded on the same gel, but lanes were re-ordered. **(D)** Immunofluorescence on meiotic nuclear spreads of myc-tagged Mre11 in wild-type and *spo11-Y135F* strains. Contrary to previously published ChIP results showing similar association and dissociation kinetics of Mre11 in wild-type and *spo11-Y135F* backgrounds[40], in our hands Mre11 foci accumulate at late time points in a *spo11-Y135F* mutant.

bioRxiv preprint doi: https://doi.org/10.1101/2025.07.08.663703; this version posted July 8, 2025. The copyright holder for this preprint (which was not certified by peer review) is the author/funder, who has granted bioRxiv a license to display the preprint in perpetuity. It is made available under aCC-BY 4.0 International license.

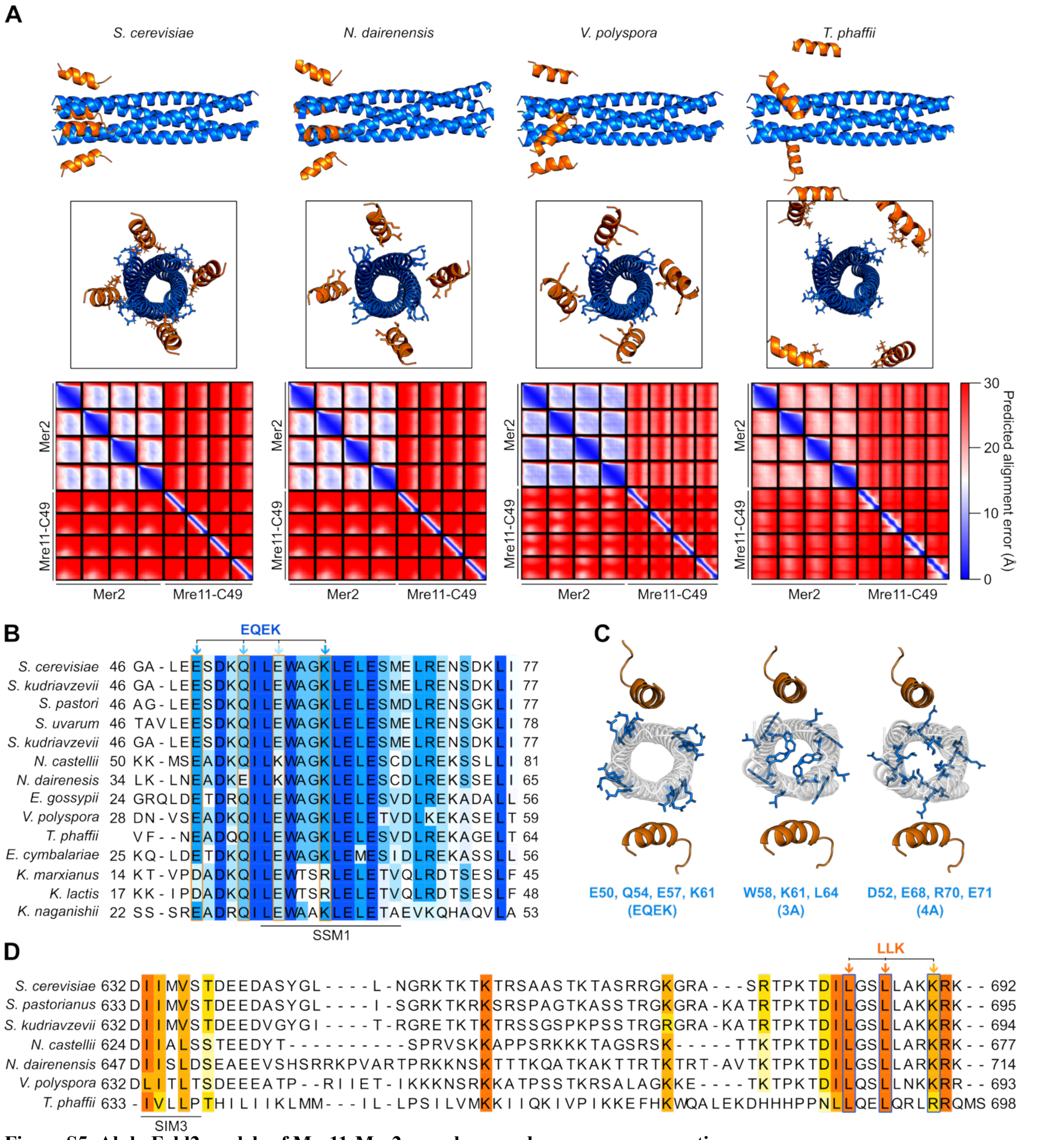

Figure S5: AlphaFold2 models of Mre11-Mer2 complexes and sequence conservation.
(A) AlphaFold2 models of 4:4 Mre11-Mer2 interaction domains in various species of Saccharomycetaceae. Mre11 is shown in orange and Mer2 in blue. Mre11-LLK and Mer2-EQEK residues are shown as orange and blue sticks, respectively, in the lateral views. Disordered regions are omitted for clarity. All models are similar, except for *T. phaffi* that is aberrant. Predicted alignment error plots for each model is shown below. Dark blue represents low predicted error and high confidence, whereas lighter shades and red indicates low confidence, typical for flexible or disordered regions. NCBI accession numbers for Mer2 and Mre11, respectively, are as follows: *Saccharomyces cerevisiae* (CAA60944, BAA02017), *Naumovozyma dairenensis* (XP_003669210.1, XP_003672532.1), *Vanderwaltozyma polyspora* (XP_001647040.1, XP_001642997.1), and *Tetrapisispora phaffii* (XP_003683996.1, XP_003686402.1). The sequences used for AlphaFold modeling are provided in **Table S5**. **(B, D)** Multiple sequence alignments of (B) Mer2 and (D) Mre11 in members of the Saccharomycetaceae class. EQEK residues are indicated by blue arrows and orange boxes and LLK residues are indicated by orange arrows and blue boxes. Alignment is colored based on percentage identity score on Jalview with a conservation threshold of 35% for Mer2 and 50% for Mre11. The previously-identified Mer2 signature sequence motif (SSM1) is indicated[44]. **(C)** Position of Mer2 EQEK residues (blue) on the AlphaFold2 model, and comparison with published 3A and 4A mutants[41]. Mre11 is in orange and Mer2 is in white.

bioRxiv preprint doi: https://doi.org/10.1101/2025.07.08.663703; this version posted July 8, 2025. The copyright holder for this preprint (which was not certified by peer review) is the author/funder, who has granted bioRxiv a license to display the preprint in perpetuity. It is made available under aCC-BY 4.0 International license.

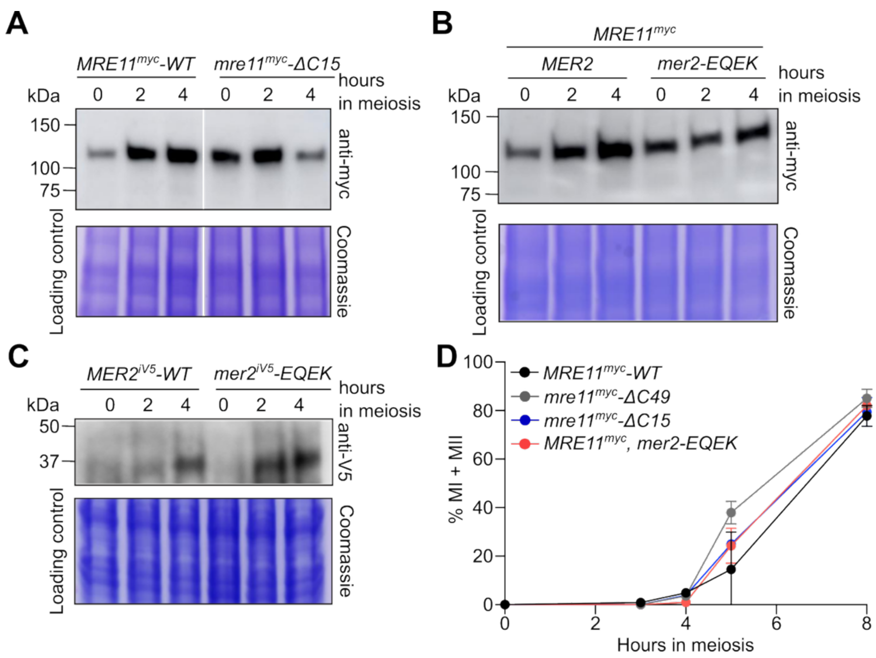

Figure S6: Analysis of Mer2 – Mre11 interaction mutants.
(A, B, C) Western blot analysis of meiotic extracts of (A) *MRE11*^{myc}*-WT* and *mre11-ΔC15*^{myc}, (B) *MRE11*^{myc} in a *MER2* or *mer2-EQEK* strain, and (C) wild type or mutant *MER2*^{iV5} strains. The *MER2*^{iV5} allele has an internal V5 tag between Mer2 amino acids 248 and 249. Coomassie-stained SDS-PAGE gels serve as loading controls. (**D**) Meiotic progression, as indicated by the percentage of cells that have undergone the first or second meiotic divisions (MI + MII) (n = 2).

bioRxiv preprint doi: https://doi.org/10.1101/2025.07.08.663703; this version posted July 8, 2025. The copyright holder for this preprint (which was not certified by peer review) is the author/funder, who has granted bioRxiv a license to display the preprint in perpetuity. It is made available under aCC-BY 4.0 International license.

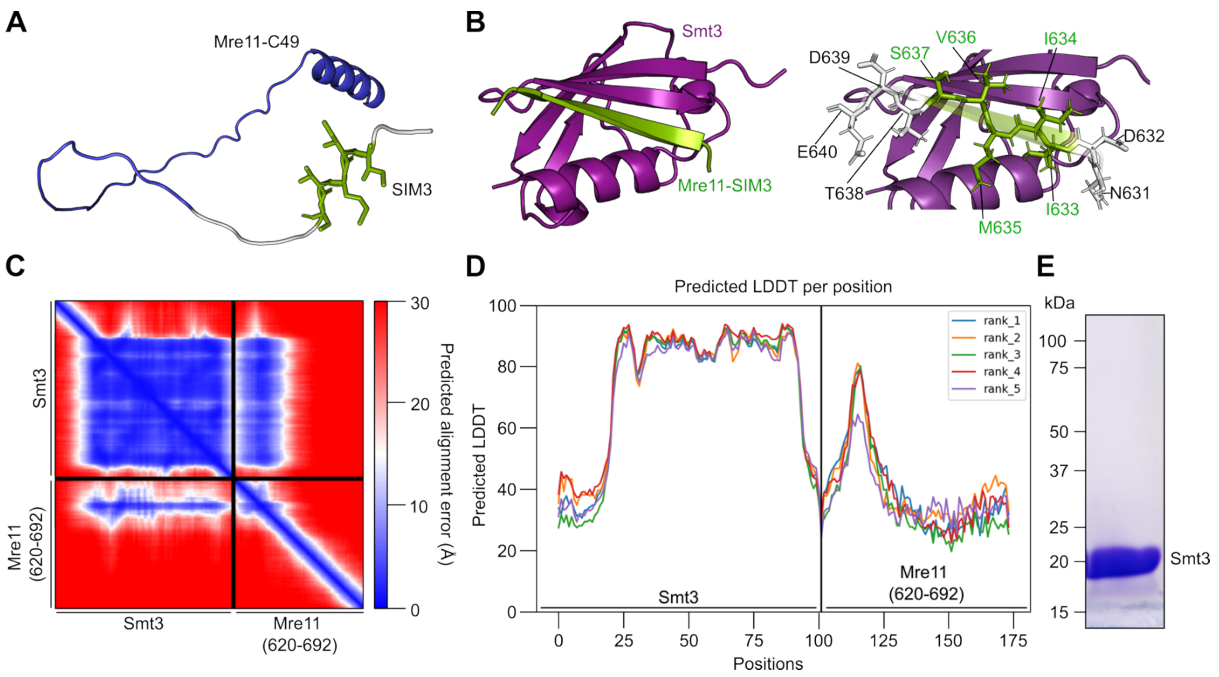

Figure S7: The Mre11 C-terminus contains a novel SUMO-interaction motif.
(A) Representation of Mre11 C-terminus (residues 630-692) from AlphaFold2 database (AF-P32829-F1-v4). The SIM3 motif (green) is located immediately before the Mre11-C49 residues (blue). **(B)** AlphaFold2 model of Smt3 (purple) and Mre11-SIM3 (residues 630-640) (green). Note that SIM3 is predicted to be disordered in panel A but folds as a β-sheet when bound to Smt3. Disordered regions of Smt3 and Mre11 are omitted for clarity. The sequences used for AlphaFold modeling are provided in **Table S5**. **(C)** Predicted aligned error (Å) plot for AlphaFold2 model of Smt3 and Mre11-SIM3 (residues 620-692). The x- and y-axis show amino acid residue numbers. Darker blue represents low predicted error and high confidence, whereas lighter shades and red indicates low confidence, typical for flexible or disordered regions. **(D)** Predicted Local Distance Difference Test (pLDDT) plot for AlphaFold2 model of Smt3 and Mre11-SIM3. The x-axis represents amino acid position and y-axis represents per-residue pLDDT score. Higher scores indicate greater confidence in local structure prediction. **(E)** SDS-PAGE of purified His-tagged U-[^{13}C,^{15}N] Smt3.

bioRxiv preprint doi: https://doi.org/10.1101/2025.07.08.663703; this version posted July 8, 2025. The copyright holder for this preprint (which was not certified by peer review) is the author/funder, who has granted bioRxiv a license to display the preprint in perpetuity. It is made available under aCC-BY 4.0 International license.

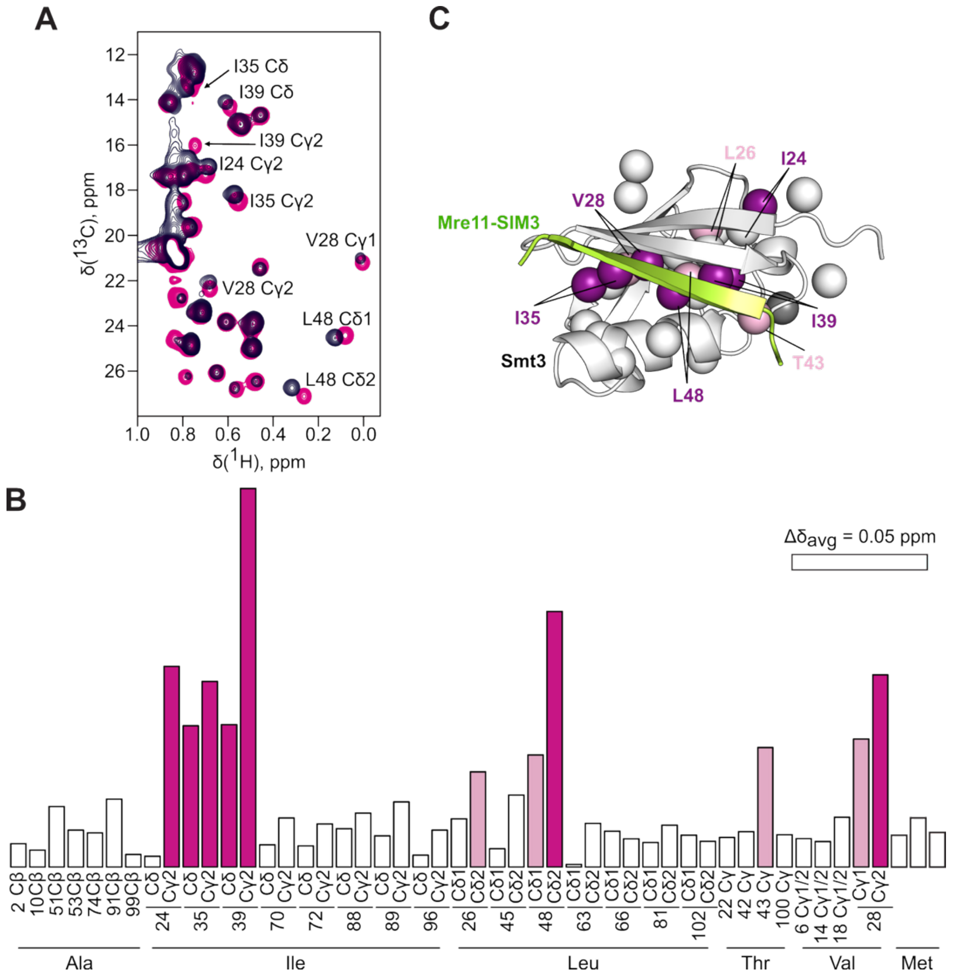

Figure S8: NMR analysis of Smt3 bound to wild type and mutant SIM3 peptides.
(A) Methyl regions of [^{1}H,^{13}C] HSQC spectra of the free Smt3 (black) and in the presence of 1.2 molar equivalents of wild type SIM3 peptide (magenta). The labels indicate the protein CH_3 groups showing the largest binding shifts. **(B)** Average methyl chemical shift perturbations ($\Delta\delta_{avg}$) of Smt3 upon binding to wild-type SIM3 peptide. The pink and magenta bars correspond to the CH_3 groups with $\Delta\delta_{avg}$ > 0.03 ppm and > 0.05 ppm, respectively. For V6, V14, and V18, which show identical Cγ1 and Cγ2 NMR resonances, a single bar is shown. As the Cδ resonances of M49, M60, and M83 were not explicitly assigned in this work, the data for the methionine methyls are represented by unmarked bars. **(C)** Chemical shift mapping of the wild type SIM3 peptide binding. Smt3 methyls are shown as spheres, colored according to $\Delta\delta_{avg}$ as in panel B. The bound SIM3 peptide is in green, and the disordered Smt3 N- and C-termini are omitted for clarity.

bioRxiv preprint doi: https://doi.org/10.1101/2025.07.08.663703; this version posted July 8, 2025. The copyright holder for this preprint (which was not certified by peer review) is the author/funder, who has granted bioRxiv a license to display the preprint in perpetuity. It is made available under aCC-BY 4.0 International license.

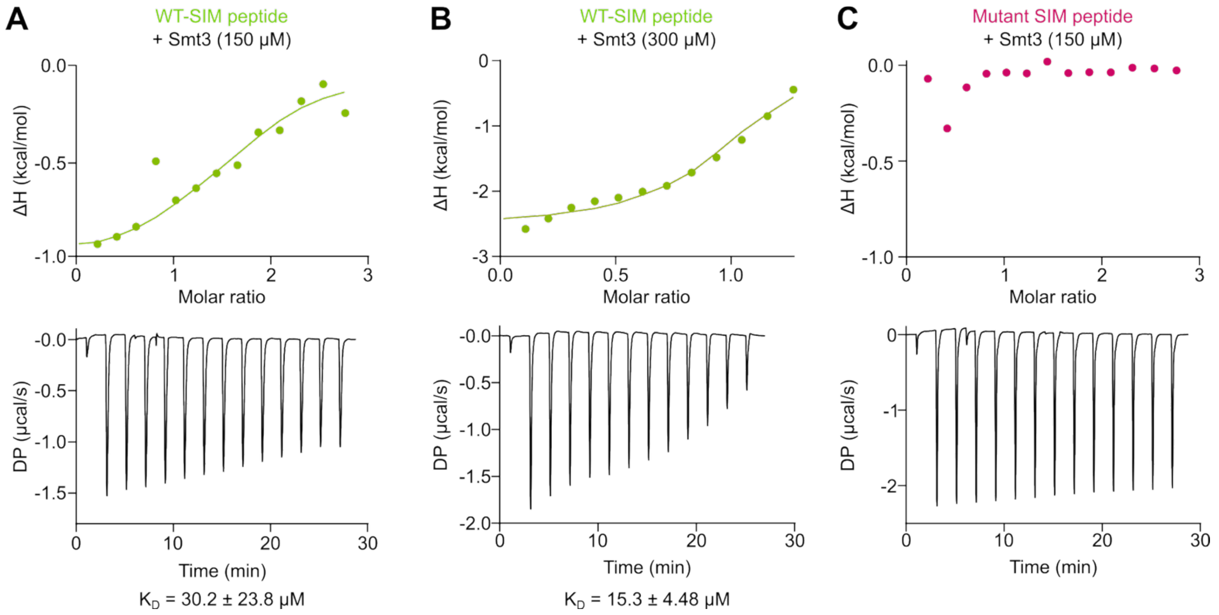

Figure S9: ITC analysis of Smt3 binding to wild-type or mutant SIM3 peptides.
Top panels, integrated heat peaks ΔH (kcal/mol) as a function of molar ratio (peptide/protein concentration) after buffer subtraction and offset correction. Bottom panels, raw data plots indicating differential power (µcal/s) after baseline correction in function of time. The data was fitted to a single binding site model and the number of binding sites (N) was set to 1. The model allowed for the calculation of the equilibrium dissociation constant K_D as provided below each graph.

bioRxiv preprint doi: https://doi.org/10.1101/2025.07.08.663703; this version posted July 8, 2025. The copyright holder for this preprint (which was not certified by peer review) is the author/funder, who has granted bioRxiv a license to display the preprint in perpetuity. It is made available under aCC-BY 4.0 International license.

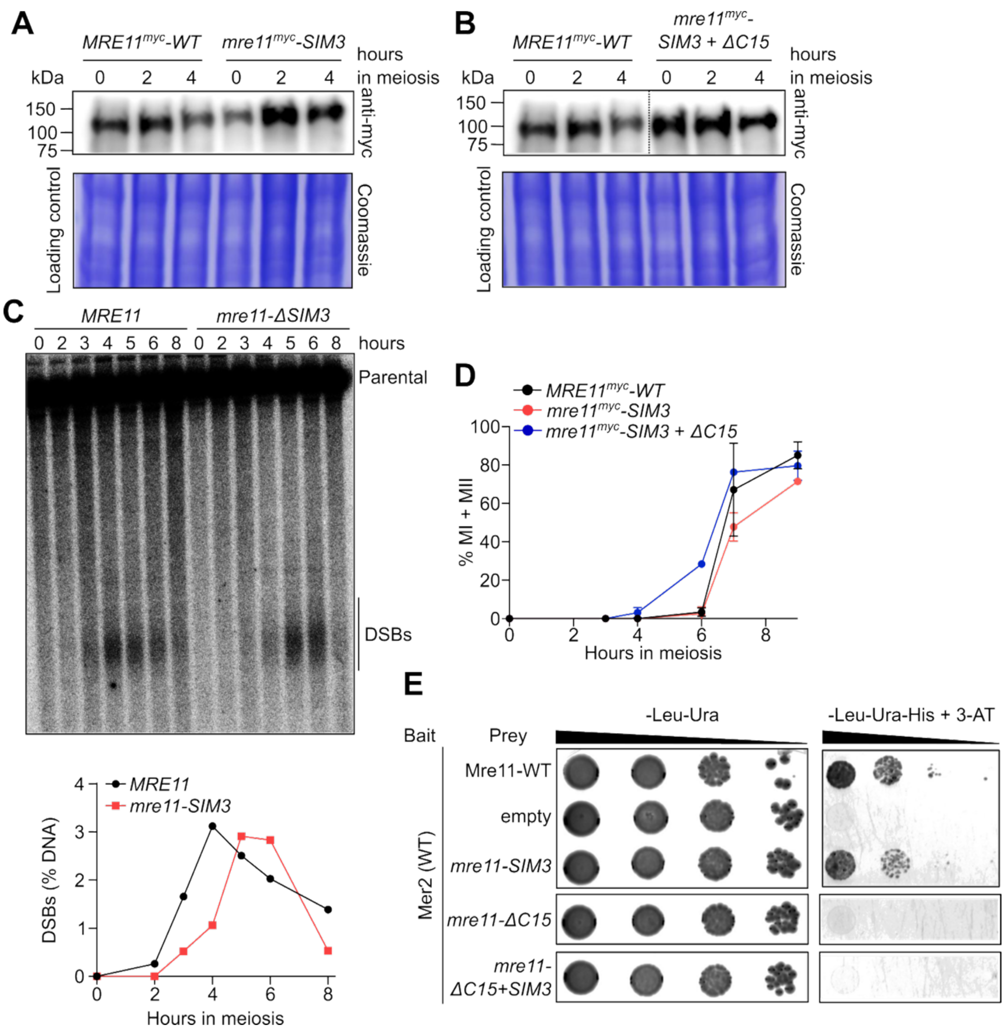

Figure S10: Protein expression and meiotic progression of Mre11 SIM3 mutants.
(A, B) Western blot analysis of meiotic extracts of (A) *MRE11*^{myc}*-WT* and *mre11-SIM3*^{myc}, (B) *MRE11*^{myc}*-WT* and *mre11-SIM3+C15*^{myc}. Coomassie-stained SDS-PAGE gels serve as loading controls. **(C)** Southern blot analysis of meiotic DSB formation at the *GAT1* hotspot. **(D)** Meiotic progression, as indicated by the percentage of cells that have undergone the first or second meiotic divisions (MI + MII) (n = 2). (**E**) Yeast-two-hybrid analysis between Mer2 wild-type (binding domain, bait) and Mre11 wild-type and mutants (activation domain, prey).

bioRxiv preprint doi: https://doi.org/10.1101/2025.07.08.663703; this version posted July 8, 2025. The copyright holder for this preprint (which was not certified by peer review) is the author/funder, who has granted bioRxiv a license to display the preprint in perpetuity. It is made available under aCC-BY 4.0 International license.

Primer No.	Sequence
cb1351	CCGTCCCACCATCGGGCGCGGATCCACCATGGACTATCCTGATCC
cb1352	GCTCGTCGACGTAGGCCTTTGAATTCCTAATGATGGTGGTGGTGATGATGATGGTGGTGTTTTCTTT TCTTAGCAAGGAGAC
cb1353	CCGTCCCACCATCGGGCGCGGATCCACCATGAGCGCTATCTATAAATTATCTATTCAG
cb1354	GCTCGTCGACGTAGGCCTTTGAATTCTCAATAAGTGACTCTGTTAATATCG
cb1355	CCGTCCCACCATCGGGCGCGGATCCACCATGTGGGTAGTACGATACC
cb1356	GCTCGTCGACGTAGGCCTTTGAATTCTCATTTGTCATCATCGTCCTTGTAATCCTTGTCATCGTCATC TTTATAATCTCCTTTTCTTCTTTTGAACG
cb1424	CGACTCACTATAGGGCGAATTGGAGCTCGAAAATAAAGGCATCTACAAATCTC
cb1425	CCTCACTAAAGGGAACAAAAGCTGGGTACCTTCAGCAAATAGCATTCAAC
cb1435	AGCGTCCTCTTCGTC
cb1458	CACCACCATCATCATCACCACCACCATCATTAGGAATTCAAAGGCCTAC
cb1486	GGATTGGAAGTACAGGTTTTC
cb1561	TATCGCCTTGTCCGATGCCTCTAAAGCGCCTGC
cb1562	CTGGCATGGGCAGGTGCACTGGAATTGGAGAGTATG
dam005	CCCTGCTGGGCCTGGACAGCACCCGTAAGAAGTCCGTGCC
dam006	GGTTGGGGATGGGCTTGCCCTGCTTATCGTGGGCT
pp3	ACTGACGAAGAGGACGC
pp4	AGCAGCTGCAGCTGCGTCATTCTCATCAATATC
pp24	AGCACGCTTAACCTGTTTTATAAGC
pp25	CCAAAGACGGATATTCTTGG
pp26	TATTGTGTCTGGATCAGGAT
pp27	GAGGCTGAGGCACAACCCAA
pp28	TGCTTCTCCATACTTTATGTC
pp29	GTAGATAAAGATGAGAAAACAGCTC
pp46	GAACAAAAGCTGGGT
pp55	CACCACCATCATCATCACCACCACC
pp59	GGCATGGACGAGCTGTACAAGGGATCCACCATGAACAGTGTTAGGCCGACTCC
pp60	GGTGGTGGTGATGATGATGGTGGTGCTTTGGCGTCCTTGATGCTCTTCC
pp61	GGTGGATCCCTTGTACAGCTCGTCCATGCC
pp72	CGTCCTTGATGCTCTTCCTTT
pp73	GAACAAAAGCTGGGTACCGG
pp84	CTAAGGCCAGAAAAATTCGAGCTCGGCGCGCC
pp85	CAAGAGCACTTCCCGCAATATCCGTCTTTGGCG
pp92	AGTTATGGTTTACTTAATGGT
pp120	CTCGAGCACCACCACCA
pp136	TGGTGGTGGTGGTGCTCGAGTCATTTTCTTTTCTTAGCAAGGA
pp148	CCATTAAGTAAACCATAACTCTTGTACAACTCATCCATAC
pp149	AAAACCTGTACTTCCAATCCATGGTGTCTAAAGGTGAAGC
RB70	AGCTGCGTCATTCTCATCAATATCAACATCATCTTG
RB267	GCAGCCGCTACTGACGAAGAGGACGCTAGTTATGG
RB268	TAAAAGCTGTCGAGAAGTACTAG
RB269	CGTCCTTGATGCTCTTCC

Table S1. Oligonucleotides used in this study.

bioRxiv preprint doi: https://doi.org/10.1101/2025.07.08.663703; this version posted July 8, 2025. The copyright holder for this preprint (which was not certified by peer review) is the author/funder, who has granted bioRxiv a license to display the preprint in perpetuity. It is made available under aCC-BY 4.0 International license.

Description	Plasmid	Reference
SUMO-Mer2 in pSMT3	pCCB750	[9]
SUMO-eGFP-Mer2 in pSMT3	pCCB777	[9]
mScarlet-Mer2 in pSMT3	pCCB785	This study
Mre11-10xHis in pFastBac1	pCCB865	This study
Rad50 in pFastBac1	pCCB866	This study
Xrs2-2xFLAG in pFastBac1	pCCB867	This study
eGFP-Mre11-10xHis in pFastBac1	pCCB942	This study
eGFP-Mre11-*ΔC49*-10xHis in pFastBac1	pCCB943	This study
Smt3-6xHis in pET28b	pCCB998	[67]
MBP-Mre11-C49 + HisSUMO-Mer2 in pETDuet1	pCCB1040	This study
Mer2-*EQEK*::HphMX in PCR-Blunt II-TOPO	pCCB1046	This study
Mer2^{iV5}-*EQEK*::HphMX in PCR-Blunt II-TOPO	pCCB1048	This study
Mre11-8xmyc::URA3 in PCR-Blunt II-TOPO	pry2	This study
eGFP-Mre11-*Δ290-472*-10xHis in pFastBac1	pry5	This study
eGFP-Mre11-*ΔIDR*-10xHis in pFastBac1	pry6	This study
eGFP-Mre11-*Δ10-270*-10xHis in pFastBac1	pry7	This study
Mre11-*ΔC49*-8xmyc::URA3 in PCR-Blunt II-TOPO	pry24	This study
Mre11-*ΔIDR*-8xmyc::URA3 in PCR-Blunt II-TOPO	pry30	This study
eGFP-Mre11-IDR-10xHis in pFastBac1	pry41	This study
Mre11-*ΔC15*-8xmyc::URA3 in PCR-Blunt II-TOPO	pry42	This study
Mre11-*SIM3*-8xmyc::URA3 in PCR-Blunt II-TOPO	pry56	This study
Mre11-*ΔC15*+*SIM3*-8xmyc::URA3 in PCR-Blunt II-TOPO	pry57	This study
MBP-Mre11-C49 + HisSUMO-Mer2-*EQEK* in pETDuet1	pry59	This study
MBP-Mre11-C49-*LLK* + HisSUMO-Mer2 in pETDuet1	pry61	This study
MBP-mScarlet-Mre11-C49 in pET28a	pry109	This study
Mer2^{iV5}::HpHMX in PCR-Blunt II-TOPO	pDAM003	This study
Mer2::HphMX in PCR-Blunt II-TOPO	pMH002	[43]
Mer2 in pGBDU-C1	pWL1592	[41]
Mre11 in pGAD-C1	pWL1596	[41]
pGAD-C1	pWL1565	[41]
Mre11-*SIM3* in pGAD-C1	pNH1371	This study
Mre11-*ΔC15* in pGAD-C1	pNH1372	This study
Mre11-*ΔC15*+*SIM3* in pGAD-C1	pNH1373	This study

Table S2. Plasmids used in this study.

Name	Sequence	Brutoformula	Calculated mass $[M+H]^+$	Measured mass $[M+H]^+$
Mre11-SIM - WT	ENDIIMVSTDE	$C_{51}H_{84}N_{12}O_{23}S$	1265.5571	1265.5593
Mre11-SIM - mutant	ENDAIAVSTDE	$C_{46}H_{74}N_{12}O_{23}$	1163.5068	1163.5074

Table S3. Synthetic peptides used in this study.

bioRxiv preprint doi: https://doi.org/10.1101/2025.07.08.663703; this version posted July 8, 2025. The copyright holder for this preprint (which was not certified by peer review) is the author/funder, who has granted bioRxiv a license to display the preprint in perpetuity. It is made available under aCC-BY 4.0 International license.

**Strain**	**Genotype**	**Reference**
CBY6	*MATa, ho::LYS2, lys2, ura3, leu2::hisG, trp1::hisG*	
CBY7	*MATα, ho::LYS2, lys2, ura3, leu2::hisG, trp1::hisG*	
CBY29	*MATα, ho::LYS2, lys2, ura3, leu2::hisG, mer2Δ::KanMX4*	
CBY317	*MATα, ho::LYS2, lys2, ura3, leu2, arg4, SPO11-Y135F-His6-flag3-loxP-hphMX-loxP*	
CBY375	*MATa, ho::LYS, ura3, leu2::hisG, trp1::hisG, MRE11-myc::URA3, arg4-nsp*	
CBY596	*MATa, ho::LYS2, lys2, ura3, leu2::hisG, trp1::hisG, MRE11-8xmyc::URA3*	This study
CBY597	*MATα, ho::LYS2, lys2, ura3, leu2::hisG, trp1::hisG, MRE11-8xmyc::URA3*	This study
CBY613	*MATα, ho::LYS2, lys2, ura3, leu2::hisG, trp1::hisG, mer2-KRRR::hphMX4*	[43]
CBY727	*MATa, ho::LYS2, lys2, ura3, leu2::hisG, trp1::hisG, arg4, MRE11-8xmyc::URA3, SPO11-Y135F-His6-flag3-loxP-hphMX-loxP*	This study
CBY728	*MATα, ho::LYS2, lys2, ura3, leu2::hisG, trp1::hisG, arg4, MRE11-8xmyc::URA3, SPO11-Y135F-His6-flag3-loxP-hphMX-loxP*	This study
CBY845	*MATa, ho::LYS2, lys2, ura3, leu2::hisG, trp1::hisG, mer2-KRRR::hphMX4, MRE11-8xmyc::URA3*	This study
CBY846	*MATα, ho::LYS2, lys2, ura3, leu2::hisG, trp1::hisG, mer2-KRRR::hphMX4, MRE11-8xmyc::URA3*	This study
CBY849	*MATa, ho::LYS2, lys2, ura3, leu2::hisG, trp1::hisG, mer2Δ::MER2*^{iV5}*::hphMX, MRE11-8xmyc::URA3*	This study
CBY850	*MATα, ho::LYS2, lys2, ura3, leu2::hisG, trp1::hisG, mer2Δ::MER2*^{iV5}*::hphMX, MRE11-8xmyc::URA3*	This study
CBY864	*MATα, ho::LYS2, lys2, ura3, leu2::hisG, trp1::hisG, mer2Δ::MER2*^{iV5}*::hphMX*	This study
CBY868	*MATa, ho::LYS2, lys2, ura3, leu2::hisG, trp1::hisG, mer2Δ::MER2*^{iV5}*::hphMX*	This study
CBY872	*MATa, ho::LYS2, lys2, ura3, leu2::hisG, trp1::hisG, mre11-ΔC49-8xmyc::URA3*	This study
CBY873	*MATα, ho::LYS2, lys2, ura3, leu2::hisG, trp1::hisG, mre11-ΔC49-8xmyc::URA3*	This study
CBY1013	*MATa, ho::LYS2, lys2, ura3, leu2::hisG, trp1::hisG, mre11-ΔIDR-8xmyc::URA3*	This study
CBY1014	*MATα, ho::LYS2, lys2, ura3, leu2::hisG, trp1::hisG, mre11-ΔIDR-8xmyc::URA3*	This study
CBY1017	*MATa, ho::LYS2, lys2, ura3, leu2::hisG, trp1::hisG, mer2-EQEK::hphMX4*	This study
CBY1018	*MATα, ho::LYS2, lys2, ura3, leu2::hisG, trp1::hisG, mer2-EQEK::hphMX4*	This study
CBY1021	*MATa, ho::LYS2, lys2, ura3, leu2::hisG, trp1::hisG, mer2*^{iV5}*-EQEK::hphMX4*	This study
CBY1022	*MATα, ho::LYS2, lys2, ura3, leu2::hisG, trp1::hisG, mer2*^{iV5}*-EQEK::hphMX4*	This study
CBY1025	*MATa, ho::LYS2, lys2, ura3, leu2::hisG, trp1::hisG, MRE11-8xmyc::URA3, mer2Δ::KanMX4*	This study
CBY1026	*MATα, ho::LYS2, lys2, ura3, leu2::hisG, trp1::hisG, MRE11-8xmyc::URA3, mer2Δ::KanMX4*	This study
CBY1027	*MATa, ho::LYS2, lys2, ura3, leu2::hisG, trp1::hisG, mre11-ΔC15-8xmyc::URA3*	This study
CBY1028	*MATα, ho::LYS2, lys2, ura3, leu2::hisG, trp1::hisG, mre11-ΔC15-8xmyc::URA3*	This study
CBY1034	*MATa, ho::LYS2, lys2, ura3, leu2::hisG, trp1::hisG, mer2-EQEK::hphMX4, MRE11-8xmyc::URA*	This study
CBY1035	*MATα, ho::LYS2, lys2, ura3, leu2::hisG, trp1::hisG, mer2-EQEK::hphMX4, MRE11-8xmyc::URA3*	This study
CBY1048	*MATa, ho::LYS2, lys2, ura3, leu2::hisG, trp1::hisG, mre11-SIM+ΔC15-8xmyc::URA3*	This study
CBY1049	*MATα, ho::LYS2, lys2, ura3, leu2::hisG, trp1::hisG, mre11-SIM+ΔC15-8xmyc::URA3*	This study
CBY1237	*MATa, ho::LYS2, lys2, ura3, leu2::hisG, trp1::hisG, mre11-ΔSIM-8xmyc::URA3*	This study
CBY1238	*MATα, ho::LYS2, lys2, ura3, leu2::hisG, trp1::hisG, mre11-ΔSIM-8xmyc::URA3*	This study
yWL365	*MATa, ura3-52, leu2-3, his3, trp1, gal4del, gal80del, GAL2-ADE2, LYS2::GAL1-HIS3, met2::GAL7-lacZ*	[41]

Table S4. Yeast strains used in this study.

bioRxiv preprint doi: https://doi.org/10.1101/2025.07.08.663703; this version posted July 8, 2025. The copyright holder for this preprint (which was not certified by peer review) is the author/funder, who has granted bioRxiv a license to display the preprint in perpetuity. It is made available under aCC-BY 4.0 International license.

Protein	Organism	NCBI Accession No.	Input sequence
Mer2	*Saccharomyces cerevisiae*	CAA60944	KVTNAGALEESDKQILEWAGKLELESMELRENSDKLIKVLNENSKTLCKSLNKFNQLLEQ
	Naumovozyma dairenensis	XP_003669210.1	SSPLKLNEADKEILKWAGKLELESCDLREKSSELIGLLKKNSTQLTSVISTLNEIVIST
	Tetrapisispora phaffii	XP_003683996.1	PVKETVFNEADQQILEWAGKLELESVDLREKAGELTTVLKRNSDRLYSVMEQLNKNLKN
	Vanderwaltozyma polyspora	XP_001647040.1	SGRDNVSEADKQILEWAGKLELETVDLKEKASELTNVFNESNTKLLELVARLNDHLGS
Mre11	*Saccharomyces cerevisiae*	BAA02017	SYGLLNGRKTKTKTRSAASTKTASRRGKGRASRTPKTDILGSLLAKKRK
	Naumovozyma dairenensis	XP_003672532.1	VARTPRKKNSKTTTKQATKAKTTRTKTRTAVTKTPKTDILGSLLARKRK
	Tetrapisispora phaffii	XP_003686402.1	ILLPSILVMKKIIQKIVPIKKEFHKWQALEKDHHHPPNLLQELQRLRRQMS
	Vanderwaltozyma polyspora	XP_001642997.1	ATPRIIETIKKKNSRKKATPSSTKRSALAGKKETKTPKTDILQSLLNKKRR
Smt3	*Saccharomyces cerevisiae*	WNF20333	MSDSEVNQEAKPEVKPEVKPETHINLKVSDGSSEIFFKIKKTTPLRRLMEAFAKRQGKEMDSLRFLYDGIRIQADQTPEDLDMEDNDIIEAHREQIGGATY
Mre11-SIM3	*Saccharomyces cerevisiae*	BAA02017 (Mre11 (residues 620-692)	NDAQDDVDIDENDIIMVSTDEEDASYGLLNGRKTKTKTRSAASTKTASRRGKGRASRTPKTDILGSLLAKKRK

Table S5. Protein sequences used for AlphaFold modeling.
